# Arm-Stroke Descriptor Variability during 200-m Front Crawl Swimming

**DOI:** 10.3390/s21020324

**Published:** 2021-01-06

**Authors:** Matteo Cortesi, Rocco Di Michele, Silvia Fantozzi, Sandro Bartolomei, Giorgio Gatta

**Affiliations:** 1 Department for Life Quality Studies, University of Bologna, 47921 Rimini, Italy; m.cortesi@unibo.it (M.C.); giorgio.gatta@unibo.it (G.G.); 2 Department of Biomedical and Neuromotor Sciences, University of Bologna, 40127 Bologna, Italy; rocco.dimichele@unibo.it (R.D.M.); sandro.bartolomei@unibo.it (S.B.); 3 Department of Electrical, Electronic and Information Engineering, University of Bologna, 40136 Bologna, Italy; 4 Health Sciences and Technologies—Interdepartmental Center for Industrial Research, University of Bologna, Via Tolara di Sopra, 50, 40064 Ozzano dell’Emilia, Italy

**Keywords:** performance, swimming technique, stroking parameters, motor adaptation, inertial sensor

## Abstract

The present study aimed to explore the variability of the arm-stroke temporal descriptors between and within laps during middle-distance swimming event using IMMUs. Eight male swimmers performed a 200-m maximum front-crawl in which the inter-lap and intra-lap variability of velocity, stroke rate, stroke-phases duration and arm-coordination index were measured through five units of IMMU. An algorithm computes the 3D coordinates of the wrist by means the IMMU orientation and the kinematic chain of upper arm biomechanical model, and it recognizes the start events of the four arm-stroke phases. Velocity and stroke rate had a mean value of 1.47 ± 0.10 m·s^−1^ and 32.94 ± 4.84 cycles·min^−1^, respectively, and a significant decrease along the 200-m (*p* < 0.001; η^2^ = 0.80 and 0.47). The end of each lap showed significantly lower stroke rate compared to the start and the middle segment (*p* < 0.05; η^2^ = 0.55). No other significant inter-lap and intra-lap differences were detected. The two main findings are: (i) IMMUs technology can be an effective solution to continuously monitor the temporal descriptors during the swimming trial; (ii) swimmers are able to keep stable their temporal technique descriptors in a middle-distance event, despite the decrease of velocity and stroke rate.

## 1. Introduction

In previous research, a variety of motion capture methods were used to assess the kinetics and kinematics of swimming. The reference method is three-dimensional video-based analysis [1,2]. Although video analysis of swimming performance has shown high accuracy and effectiveness, it has some limitations like short capture periods, elaborate setting process and extensive data processing [3,4]. Recently, the use of inertial and magnetic measurement unit (IMMU) technology based on inertial, magnetic sensors and gyroscopes, has gained much interest in sports applications, including swimming analysis [5,6]. Indeed, IMMU systems allow one to continuously monitor the swimming action without spatial limitations and they do not require long-time post-processing or complex experimental setup [7,8].

Swimming velocity (*v*) is a key factor when assessing swimming performance. Velocity is determined by examining several stroke cycles and can be described by stroke length (SL) and stroke rate (SR). During a front crawl race, the decrease of *v* is related to almost steady or slightly increasing SR [9,10]. According to Figuereido et al. [11], in a 200-m front crawl race, the inter-lap SR develops a U-shaped manner with a smooth increase at the end of the effort, to compensate the decrease in SL due to augmented effort when attempting to maintain a constant speed. Thus, the swimmers adjust *v* by adapting their SR, since an almost even pace is optimal for 200-m swimming performance across all strokes [12].

The stroke cycle in swimming, according to Chollet et al. [13], can be described by four distinct arm-stroke phases: entry, pull, push, and recovery. The duration of each phase varies from one swimmer to another and many investigations showed that swimmers adjust the time spent in each stroke phase for performance purposes [9]. The swimming stroke in long-distance swimming events is characterized by an extended entry phase and a decreased duration of the propulsive phases in comparison to sprint events [14]. During short events, swimmers increase the time spent in the pull and push (propulsive) and reduce the relative duration of the entry and recovery phases (non-propulsive). Thus, the swimmer can maximize the propulsive time of the arm-stroke phase to improve his/her performance. In general, arm-stroke phases durations are characterized by a pattern of low inter-lap variability, as shown by Schnitzler et al. [15] for distances of 400 m. However, Seifert et al. [16] highlighted that swimmers exhibiting medium and low speed spend significantly longer time in the push phase during the last lap of a maximal 100-m front crawl, although the reduction of SL makes ineffective such adjustment. Nevertheless, until a decade ago, the methods and instruments used for assessing the stroke cycle in swimming allowed to measure technical descriptors for only few cycles.

An additional temporal variable aimed to understand the technique modifications under fatigue condition is the Index of Coordination, which was largely used as a technique model of inter-limb coordination (IdC) [13]. In front crawl, the IdC is based on the measurement of the lag time between the propulsive phases of the two upper limbs. Inter-lap results in a 200-m all-out test showed an increase in the relative duration of the propulsive phase, suggesting a compensation for the decline of the force generating capacity [17]. Consequently, a modification in inter-limbs coordination occurred as effort increased. Similar results were reported by Seifert et al. [18] for a 100-m test, who reported a change of the IdC between the beginning and the end of a race. However, the IdC over the eight laps of a 200-m trial stayed within the catch-up model of arm coordination, where a lag time exists between the propulsive phases of the two arms [19]. In this context, the spatio-temporal parameters seem key features in the approach to understand the structure of the variability of swimming performance [20].

A way to understand how a cyclic movement like swimming may be optimized to achieve the highest possible performance, is to assess the variability characterization of technique descriptors [20]. Thus, the use of wearable IMMUs offers interesting perspectives for a more comprehensive representation of the swimming technique. To address the issue of the variability of technical parameters, the results of Cortesi et al. [1] and Dadashi et al. [21] confirmed the validity of IMMUs technology in the analysis of multi-cycle kinematics variability for the stroke phases duration and IdC. Using IMMUs, Seifert et al. [22] highlighted higher inter-individual variability for temporal descriptor and suggested that arm-stroke variability is not necessarily detrimental to achieve high performance. However, the only study that described the multi-cycle stroke variability within and between lap for stroking temporal parameters using IMMUs was that of Dadashi et al. [23], highlighting a stable pattern of stroke phase duration and IdC in swimming trials performed at submaximal intensity. Those results suggested the necessity of an investigation of changes of arm-stroke variability for stroking temporal parameters with augmented effort since no study has focused on multi-cycle kinematics within and between lap variability in a 200-m swimming trial performed at maximal intensity. For these reasons, the aim of this study was to investigate, using IMMUs, the inter-lap (between lap) and intra-lap (within lap) variability of the arm-stroke temporal descriptors in an all-out 200-m front crawl. We hypothesized a stable pattern for these descriptors across swim trials and laps despite the increase in effort.

## 2. Materials and Methods

### 2.1. Design and Participants

To test the hypothesis, an observational study with one data collection session for any participant was designed. We conducted the study in a 25-m indoor swimming pool (average water temperature: 28.0 ± 0.5 °C) over a period of two months (October and November) at morning time (9:00–12:00). Each swimmer spent an average of 2 h at the pool. The experimental trial consisted of performing a 200-m maximum front crawl in which the variability of arm-stroke parameters (*v*, SR, stroke phases duration and IdC) was measured using IMMUs.

Eight well-trained male swimmers (22.0 ± 2.5 years of age; 75.6 ± 6.3 kg of body mass; 1.78 ± 0.05 m of stature) volunteered to participate in this study. The participants had an average of 11.9 (± 3.5) years of competitive experience, a weekly training duration of 12 ± 2 h, training volume of 40–45 km and average performance in the 200-m short-course front crawl swim of 122.7 ± 3.7 s (representing 74.8 ± 3.8% of the World Record). The inclusion criteria were: (i) more than 10 h of swimming training per week; (ii) personal best in the 200-m short-course front crawl swim equaling more than 70% of the World Record. Participants were members of a competitive swimming team who compete in regional and national swimming events. This sample can be considered as a representative of well-trained swimmers. Participants were excluded if they presented any relevant neuromuscular-musculoskeletal injury or reported to have performed extenuating exercise in the 48 h previous to any assessment.

The project was approved by the local Bioethics Committee (Approval code: 0196686) and conducted according to the ethical standards of the Declaration of Helsinki. All subjects were properly informed about the study purpose, and a written consent was obtained before any formal testing.

### 2.2. Measuring Protocol

The swimmers performed a 200-m front crawl simulated race at maximal intensity from a push-off start (the dive was not performed to not influence the analysis of the first stroke cycle). Data collection was performed using five IMMUs (APDM Opals, Portland, OR, USA, 128 Hz with internal storage 8 Gb), including tri-axial gyroscopes (±2000°/s), tri-axial magnetometers (±6 gauss) and tri-axial accelerometers (±6 g) each. The weight of each unit was <25 g, including the battery. Before data collection, the five IMMUs were calibrated as described by Cortesi et al. [1]. Each unit was inserted in round plastic waterproofed boxes and then fixed to the swimmer’s body segments at the level of thorax, upper-arms, and fore-arms by means of adhesive tape/spray and elastic bands. Before the experimental trial, the swimmers completed an individual warm-up, for a total of 1000 m, and performed a 50-m front crawl trial at submaximal pace wearing IMMUs to become familiar with the protocol set-up.

**Figure 1:**
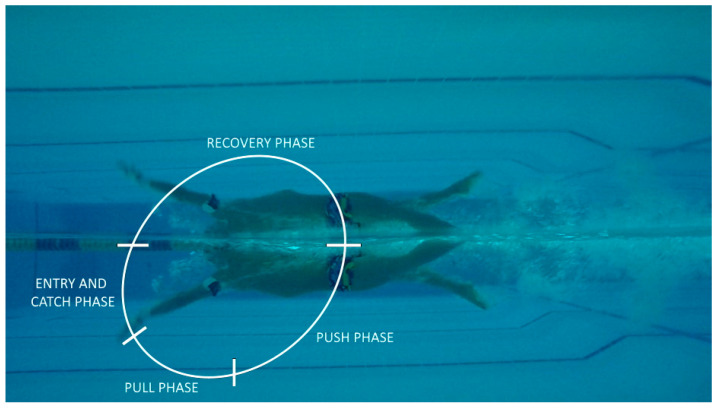
Graphical presentation of the arm-stroke phases classification proposed by Chollet [13] plotted in a single frame captured by underwater camera during the experimental condition.

The automatic recognition of the start events of the four arm-stroke phases during 200-m was performed using an algorithm previously validated, that computes the 3D coordinates of the wrist by means the orientation of the inertial sensor and the kinematic chain of the upper arm [1]. Repeatability and reliability of the proposed algorithm between arm-stroke cycles was verified by previous analysis [1]. The same arm-stroke phases classification of Chollet et al. used in previous research with IMMUs [21] was defined: (i) the entry and catch phase corresponded to the time between the hand’s entry into the water and the beginning of its backwards movement (t_ENTRY_); (ii) the pull phase represented the beginning of the propulsion and corresponded to the time from the beginning of the hand’s backwards movement until the hand entry to the transversal plane crossing the shoulders (t_PULL_); (iii) the push phase corresponded to the time from the hand’s position below the shoulder to its release from the water (t_PUSH_); the recovery phase corresponded to the time from the hand’s release from the water to its subsequent entry into the water (t_RECOVERY_). Each phase ended with the start of next one, and all the arm-stroke phases duration were expressed as a percentage of the complete duration of the stroke cycle (t_ENTRY_%, t_PULL_%, t_PUSH_% and t_RECOVERY_% for entry and catch, pull, push and recovery phases, respectively). See Figure 1 for details.

Arm coordination was quantified using the IdC according to one of three major models: (i) catch-up describing a time delay between the propulsive phases of the two arms (IdC < 0); (ii) opposition explaining a propulsive action if the propulsive phase of one arm started when the other arm ended (IdC = 0); (iii) superposition describing an overlap of the propulsive phases (IdC > 0).

The above described procedures enabled to calculate the stroke frequency as the time needed to complete a stroke cycle using t_ENTRY_ as a time reference. Then, SR was calculated by dividing 60 s by stroke frequency. The proposed algorithms in literature based on IMMUs do not allow to accurately calculate the instantaneous velocity of the swimmer during a non-brief event. Indeed, the accurate determination of the instantaneous swimming velocity using IMMUs is a current area of research to solve the actual relative errors in velocity estimation, in the drift of the acceleration data integration, and in the versatility to individual movement characteristics [5,8]. Therefore, the intra-lap variability of *v* and SL were not calculated.

Lap time was directly measured by underwater video cameras (GoPro Hero 7, GoPro, San Mateo, CA, USA) recording at 120 Hz and full HD resolution (1920 × 1080 pixel) and anchored on a trolley to record the athlete along the sagittal plane. The trolleys were pulled by an operator at the swimmer’s head level at the same velocity of the swimmers. Each lap contained a minimum of seven and a maximum of nine arm-stroke cycles. Each lap was divided into three lap segments for intra-lap variability calculation: the first segment contained the first two or three arm-stroke cycles of the lap (LAP_start_); the second segment contained the second two or three arm-stroke cycles of the lap (LAP_mid_); the third segment contained the last two or three arm-stroke cycles of the lap (LAP_finish_). The segment partition, based on the number of stroke cycles and not on the area of the lane, was chosen to standardize the number of cycles per segment as much as possible, avoiding the influence of the underwater phase.

### 2.3. Statistics

Normal distribution of data was verified by Shapiro–Wilk tests and sphericity was checked using Mauchly tests. The dependent variables were compared using analysis of variances (ANOVAs) for repeated measures to investigate the effects of changes over the eight laps (inter-lap variability) and the segments of the lap (intra-lap variability). The possible lap conditions were 1, 2, 3, 4, 5, 6, 7, or 8, where lap segment conditions were LAP_start_, LAP_mid_ and LAP_finish_. When a significant main effect was found, the Fisher’s LSD post hoc test was carried out. Effect sizes were computed by the eta-squared (η^2^) and interpreted as: without effect if 0 < η^2^ ≤ 0.04; minimum if 0.04 < η^2^ ≤ 0.25; moderate if 0.25 < η^2^ ≤ 0.64 and; strong if η^2^ > 0.64. All data were expressed as means (±SD). All statistical tests were performed using the software SPSS version 20.0 (SPSS, Chicago, IL, USA). Statistical significance was set at *p* < 0.05.

## 3. Results

**Figure 2:**
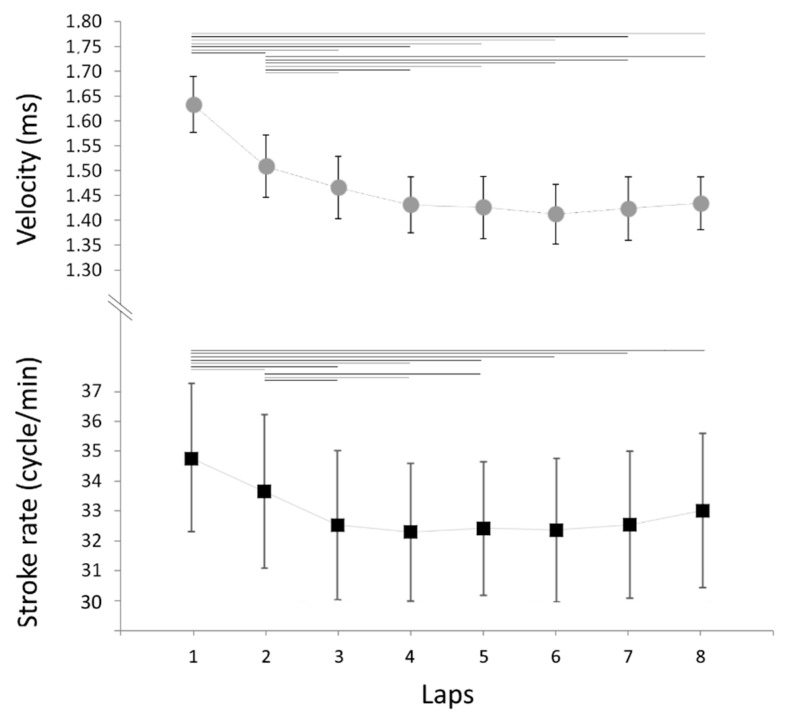
Mean ± SD values for v (filled grey circles) and SR (filled black squares) as a function of 25-m laps (inter-lap variability). Horizontal lines indicate significant differences (*p* < 0.05).

Mean ± SD relative values of *v* and SR for each lap are presented in Figure 2. The results showed differences for laps condition with strong effect sizes. The ANOVA indicates a significant decrease of *v* along the 200-m (F_7.49_ = 28.232; *p* < 0.001; η^2^ = 0.80, strong). The first and second lap showed a higher *v* than the remaining laps (*p* < 0.05). SR had a mean value over whole trail of 32.94 ± 4.84 cycles⋅min^−1^ and a significant decrease along the 200-m (F_7.49_ = 6.133; *p* < 0.001; η^2^ = 0.47, moderate): the first lap showed a significantly higher SR than the other laps; the second lap showed a significantly higher SR than the other except lap 6, 7 and 8 (*p* < 0.05, see Appendix A for post hoc test results). No other difference in *v* and SR was found between laps.

**Figure 3:**
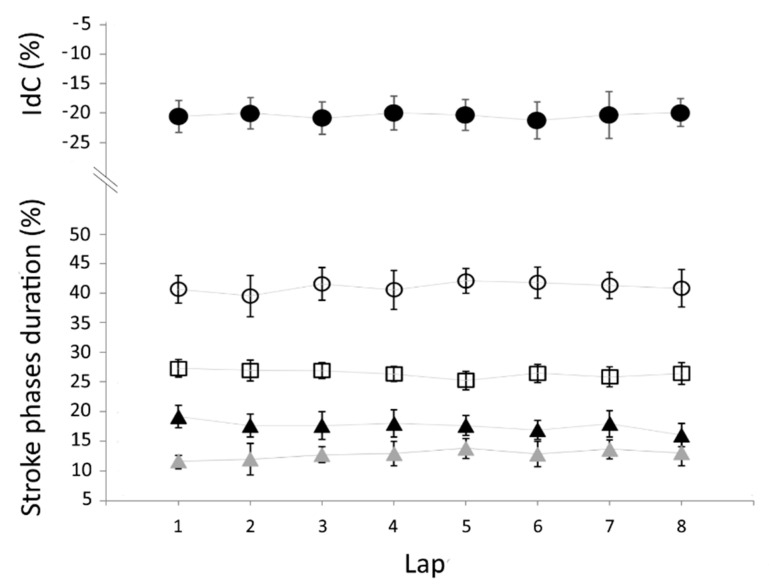
Mean ± SD values in percentage of index of coordination (filled black circles) and stroke phases duration (t_ENTRY%_ = empty circles; t_PULL%_ = filled black triangles; t_PUSH%_ = filled grey triangles; t_RECOVERY%_ = empty squares) are plotted as a function of the 25 m laps (inter-lap variability).

Mean ± SD relative durations of stroke phases duration and IdC over the eight laps of the 200-m are reported in Figure 3. A stable pattern along the eight laps of the 200-m trial (inter-lap variability) is confirmed by the non-significant difference for relative duration of each stroke phase: t_ENTRY%_ (F_7,49_ = 0.751; *p* = 0.63; η^2^ = 0.10), t_PULL%_ (F_7,49_ = 1.661; *p* = 0.14; η^2^ = 0.19), t_PUSH%_ (F_7,49_ = 1.002; *p* = 0.44; η^2^ = 0.13) and t_RECOVERY%_ (F_7,49_ = 0.635; *p* = 0.73; η^2^ = 0.08). There was no main effect for laps condition also for the IdC (F_7,49_ = 0.100; *p* = 0.99; η^2^ = 0.14).

**Figure 4:**
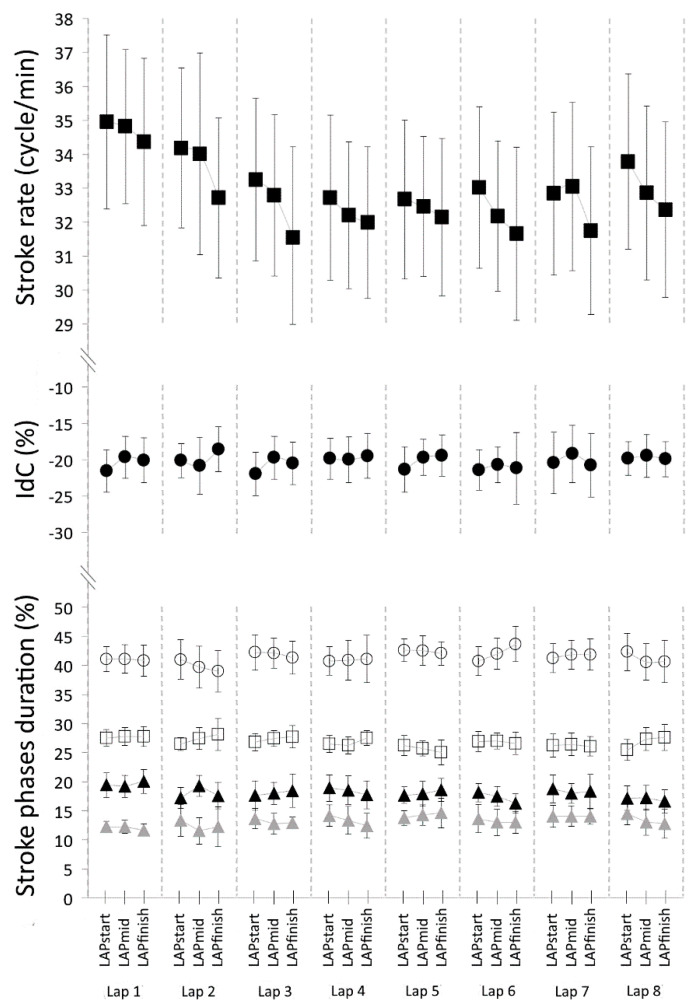
Mean ± SD values of SR (filled black squares), index of coordination (filled black circles) and stroke phases duration (t_ENTRY%_ = empty circles; t_PULL%_ = filled black triangles; t_PUSH%_ = filled grey triangles; t_RECOVERY%_ = empty square), plotted as a function of lap segment (LAP_start_, LAP_mid_ and LAP_finish_) of the 200-m trial (intra-lap variability).

Results of the ANOVA for lap segment condition (intra-lap variability) are shown in Figure 4. Two-way ANOVAs revealed significant differences in the SR for laps segment condition (F_2,14_ = 8.401; *p* < 0.05; η^2^ = 0.55, moderate), between LAP_start_ and LAP_finish_, and between LAP_mid_ and LAP_finish_. No significant lap segment differences were found for the others variables: IdC (F_2,14_ = 4.493; *p* = 0.31; η^2^ = 0.39 η^2^), t_ENTRY%_ (F_2,14_ = 0.130; *p* = 0.88; η^2^ = 0.02 η^2^), t_PULL%_ (F_2,14_ = 0.972; *p* = 0.40; η^2^ = 0.12), t_PUSH%_ (F_2,14_ = 3.159; *p* = 0.07; η^2^ = 0.31 η^2^) and t_RECOVERY%_ (F_2,14_ = 1.011; *p* = 0.39; η^2^ = 0.13). There was no significant lap by lap segment interaction for all the variables: SR (F_14,98_ = 8.858; *p* = 0.61; η^2^ = 0.11), IdC (F_14,98_ = 0.607; *p* = 0.85; η^2^ = 0.08 η^2^), t_ENTRY%_ (F_14,98_ = 1.379; *p* = 0.18; η^2^ = 0.16 η^2^), t_PULL%_ (F_14,98_ = 1.097; *p* = 0.37; η^2^ = 0.13), t_PUSH%_ (F_14,98_ = 0.764; *p* = 0.70; η^2^ = 0.10 η^2^) and t_RECOVERY%_ (F_14,98_ = 0.943; *p* = 0.52; η^2^ = 0.12).

## 4. Discussion

The present study aimed to explore the variability of the arm-stroke temporal descriptors between and within laps during middle-distance swimming event using IMMUs. The findings show a stable pattern with no difference across the eight laps of the 200-m for stroke phases duration and IdC, despite a significant decrease of *v* and SR along the trial. Furthermore, the present results indicate that swimmers can maintain stability within each lap, with exception of SR, that seems to decrease at the end of each lap. This information can assist swimming-related professionals in determining the optimal strategy for arm-stroke temporal descriptors during middle-distance swimming events. The findings also suggested that IMMUs technology can be an effective solution to monitor the arm-stroke temporal descriptors continuously during the whole swimming trial.

In swimming races, the patterns of effort distribution over an exercise (pacing) should focus on optimal individual strategy, because swimmers are not in close physical proximity [24]. The forces of drag and friction seem key determinants of different pacing models between competitive sports activities. 200-m swimmers, who experienced the highest drag, keep to an even pace in comparison to 800-m runners and 1500-m speed skaters that start faster. Nevertheless, the most commonly used pacing strategies in 200-m swimming are parabolically shaped, with a fast start followed by an evenly paced mid-section and a fast end sprint, or only fast-start even [12,25]. Similarly, in our study, the first and second lap showed a higher *v* than the remaining laps, confirming the fast-start even pacing model observed in the 200-m front crawl events at the 2013 World Swimming Championships, where the swimmer’s velocity remained stable in the last laps of the race [26]. Consistently, Simbana-Escobar et al. [10] confirmed the highest speed was that of the first lap of a 200-m front crawl event in comparison to the other laps. This stable velocity during the middle laps can be explained considering what happens in the underwater phase, as the underwater velocities after start and turn are not meaningfully affected by the race progress, as showed previously by Veiga et collaborators [26], thus allowing swimmers to maintain their average velocity in the last laps.

Swimming speed is the product of SR and SL, where SR was highlighted to contribute most to achieve high swimming speeds [9]. In a 200-m front crawl, high swimming speeds are related with high SR as demonstrated by other authors [17,27]. This is consistent with findings of our investigation, where the first two laps were performed with higher SR than the following laps. Indeed, an increase in stroke rate can lead to an increase in swimming speed and performance [28]. These findings suggest and support that the management of the stroking parameters is a key aspect of performance that coaches should consider when designing training. However, the only analysis of between laps variability might implicate the impossibility to accurately determine the arm-stroking management during a race. The ability to keep a constant stroke throughout the multi-cycles of a 200-m is an essential skill of able swimmer [29]. The intra-lap results of the present study indicate a variable behaviour of SR for the laps segment condition, with a significant reduction at the end of each lap in comparison to the first and middle segments. Previously, Seifert et al. [18] and Hellard et al. [27] quantified the SR cycle-by-cycle in a 200-m front crawl and showed similar results with lower variability in SR within laps and lower values of SR during the finish of laps after the turns. Such behavior seems strongly related to the lower speed at the end of each lap of the 200-m highlighted by Simbana-Escobar et colleagues [10], probably due to neuromuscular fatigue of the continuous cyclic action. Similarity, Seifert et al. [16] highlighted that the changes in the finish of lap can be attributed to fatigue. This could suggest that intra-lap stroke rate variability is speed-dependent, and the turns play an important role in the recovery of the strenuous cyclic action. The 200-m front crawlers seem to be able to rapidly increase their *v* and SR after the turn to better adapt to the task constraints of the race. Therefore, it is recommended that swimming coaches consider the impact of turning phases on arm-stroking management.

Despite the reduction of *v* and SR due to increasing effort, the sprinter in swimming exhibit good stability in their stroke phases duration and IdC [16]. The stability of stroke phases duration for swimming distances with major aerobic contribution, and especially the 400-m front crawl, was shown by Schnitzler et collaborators [15]. Although they collected only three stroke cycles every 50-m, the absolute values of stroke phases duration were consistent with our results, with a propulsive phase range of 30–35%. The findings of the present study confirm that no variation occurs in the relative duration of each stroke phase along the eight laps of the 200-m maximal trial, supporting the high capacity of well-trained swimmers to standardize their motor pattern as shown by Dadashi et al. [23] at submaximal intensities. On the contrary, for the 100-m front crawl, where the anaerobic pathway is the major contributor to energy production, previous investigations reported that sprinters spent more time with the hand in the push phase probably for an effect of fatigue [16]. Furthermore, supporting the stability of arm-stroke temporal descriptors in the middle-distance swimming events, the present results demonstrated no differences between the first, middle and last segment of the lap for phases duration. This kind of intra-lap stability had never been shown previously for all-out 200-m trials, but only for submaximal trials. Therefore, it appears that skilled swimmers are able to avoid the technique degradation due to the augmented effort more effectively for coordination parameters than SR.

In this scenario, the behavior of the IdC is similar to that in the above observations. The swimmers in the present study maintained strong stability of IdC model between and within each lap. A similar pattern for inter-lap variability of IdC with no significant differences between laps was reported by Schnitzler et collaborators [15] for efforts mostly supported by the aerobic system of energy production. On the other hand, Figueiredo et al. [17] and Alberty et al. [19] showed variations in inter-limbs coordination from the first to the last lap of a 200-m front crawl due to an increase in the relative duration of the propulsive phase, but the stability of the catch-up model at the same negative absolute values of our study was confirmed. Supposedly, the discrepancy between the first two studies and the present one is due to the low number of stroke cycles that can be captured with a video-based system in the former studies. This study confirmed that expert swimmers are able to keep relative stability of their arm-stroke temporal descriptors postponing a technique degradation, conversely to swimmers of less expertise as shown by Seifert et al. [16]. Hence, skilled swimmers demonstrate to adapt their perceptions/actions and coordination strategy to limit the loss of speed and to adapt to the environmental constraints and tasks [20]. Future studies are warranted to investigate the role of different pacing strategies on the variability of arm-stroke temporal descriptors in skilled swimmers for a more comprehensive characterization of the technique degradation due to fatigue.

A limitation of this study concerns the non-inclusion of SL in the temporal parameters analyzed. However, underwater velocities and the travelled distance in skilled 200-m front crawlers stay stable throughout the laps, despite a decrease in the free-swimming velocity [26]. Consequently, if the underwater distance remains stable during the trial, SL can be calculated from the average *v* and SR. Such calculation, nevertheless, would not have allowed estimating the within lap variability of the temporal parameters. On the contrary, an advantage of the approach used in the present study is the possibility of tracking the temporal parameters at every swim-stroke cycle and consider therefore the effort effects on these parameters. Thus, this study points out the relevance of multi-cycle data acquisition in the analysis of the arm-stroke temporal descriptors, in order to avoid possible misrepresentative results due to the low number of stroke cycles captured with a video-based system.

## 5. Conclusions

This study aimed to analyze the variability of the arm-stroke temporal descriptors during a maximal effort with a prevalent aerobic contribution, in order characterize technical parameters. The present finding indicate that swimmers are able to keep stable their temporal technique descriptors in a middle-distance swimming event, despite the decrease of v and SR. To the best of our knowledge, this is the first study that highlights the ability of skilled swimmers to reproduce the same stroke phases duration and IdC between and within laps of a maximal 200-m front crawl for all stroke cycles. The results indicate that IMMU technology can provide informative measures to recognize the variability of temporal technique descriptors during middle-distance swimming events. This also suggests that the management of the variability of arm-stroke temporal parameters may have a key role in the swimming technique monitoring.

## Data Availability

The data presented in this study are available on request from the corresponding author. The data are not publicly available due to privacy restrictions.

## Appendix A

**Table A1:** Summary of significant results of ANOVA and Fisher’s LSD.

Source of Variation	df	Mean Square	*F*	η^2^	*p*	Mean Difference	Lower Bound 95% CI	Upper Bound 95% CI
**SR**								
Between laps	7, 49	17.405	6.133	0.467	<0.001 *			
1–2					<0.001 *	1.115	0.67881	1.55162
1–3					<0.001 *	2.212	1.28028	3.14426
1–4					<0.001 *	2.43	1.47499	3.38492
1–5					<0.001 *	2.274	1.25367	3.29474
1–6					<0.05 *	2.441	1.04285	3.83877
1–7					<0.05 *	2.185	0.67050	3.69899
2–3					<0.05*	1.097	0.14479	2.04933
2–4					<0.05 *	1.315	0.31094	2.31854
2–5					<0.05 *	1.159	0.13492	2.18307
Within laps	2, 14	20.308	8.401	0.545				
LAP_start_-LAP_finish_					<0.05 *	1.105	0.25282	1.95697
LAP_mid_-LAP_finish_					<0.05 *	0.743	0.12614	1.36000
*v*								
Between laps	7, 49	0.0435	28.232	0.801	<0.001 *			
1–2					<0.001 *	0.124	0.08821	0.16008
1–3					<0.001 *	0.167	0.11684	0.21666
1–4					<0.001 *	0.202	0.14236	0.26111
1–5					<0.001 *	0.207	0.13214	0.28123
1–6					<0.001 *	0.221	0.15715	0.28388
1–7					<0.001 *	0.209	0.11992	0.29809
1–8					<0.001 *	0.198	0.13238	0.26383
2–3					<0.05 *	0.043	0.00854	0.07666
2–4					<0.05 *	0.078	0.03054	0.12464
2–5					<0.05 *	0.083	0.02993	0.13514
2–6					<0.05 *	0.096	0.04871	0.14402
2–7					<0.05 *	0.085	0.01294	0.15677
2–8					<0.05 *	0.074	0.01821	0.12971
3–4					<0.05*	0.035	0.01489	0.05509
3–5					<0.05 *	0.040	0.01323	0.06665
3–6					<0.05 *	0.054	0.03079	0.07674
3–8					<0.05 *	0.031	0.00321	0.05951
6–8					<0.05 *	−0.022	−0.03859	−0.00623

* Significant difference.
